# A Compact Microelectrode Array Chip with Multiple Measuring Sites for Electrochemical Applications

**DOI:** 10.3390/s140609505

**Published:** 2014-05-28

**Authors:** Maria Dimaki, Marco Vergani, Arto Heiskanen, Dorota Kwasny, Luigi Sasso, Marco Carminati, Juliet A. Gerrard, Jenny Emneus, Winnie E. Svendsen

**Affiliations:** 1 DTU Nanotech, Technical University of Denmark, Oersteds Plads, Bldg 345E, 2800 Kgs. Lyngby, Denmark; E-Mails: arto.heiskanen@nanotech.dtu.dk (A.H.); dorota.kwasny@nanotech.dtu.dk (D.K.); jenny.emneus@nanotech.dtu.dk (J.E.); winnie.svendsen@nanotech.dtu.dk (W.E.S.); 2 Dipartimento di Elettronica e Informazione, Politecnico di Milano, Piazza Leonardo da Vinci 32, I-20133 Milano, Italy; E-Mails: Marco.Vergani@flextronics.com (M.V.); marco.carminati@polimi.it (M.C.); 3 The MacDiarmid Institute for Advanced Materials and Nanotechnology, Biomolecular Interaction Centre, School of Biological Sciences, University of Canterbury, Private Bag 4800, Christchurch 8140, New Zealand; E-Mails: luigi.sasso@canterbury.ac.nz (L.S.); juliet.gerrard@canterbury.ac.nz (J.A.G.)

**Keywords:** microfabrication, lift-off ears, electrochemical applications, multiple measuring sites

## Abstract

In this paper we demonstrate the fabrication and electrochemical characterization of a microchip with 12 identical but individually addressable electrochemical measuring sites, each consisting of a set of interdigitated electrodes acting as a working electrode as well as two circular electrodes functioning as a counter and reference electrode in close proximity. The electrodes are made of gold on a silicon oxide substrate and are passivated by a silicon nitride membrane. A method for avoiding the creation of high edges at the electrodes (known as lift-off ears) is presented. The microchip design is highly symmetric to accommodate easy electronic integration and provides space for microfluidic inlets and outlets for integrated custom-made microfluidic systems on top.

## 1. Introduction

In recent years there has been a dramatic increase in the number of reported lab-on-a-chip systems utilizing electrochemical techniques for the detection of a wide range of analytes, e.g., DNA, amino acids, peptides, drugs and even explosives [1]. The main reason for this increased interest is that electrochemical detection can easily be integrated on-chip, since it only requires the manufacturing and miniaturization of electrodes for which conventional microfabrication methods can be used. Moreover, the method is reliable, providing high sensitivity [2], as well as a high degree of specificity and selectivity, when appropriate modifications are applied. One other advantage is that the potential required to perform electrochemical analysis is low opening up the possibilities of developing portable battery operated point-of-care systems [3].

Electrochemical sensing is usually carried out in a 3-electrode system comprising a working electrode (WE) where the reactions of interest take place, a reference electrode (RE) in proximity to the WE to maintain a known and stable potential, and a counter electrode (CE) to facilitate electron transfer that is complementary to the processes taking place at the WE allowing the resulting current to pass through the electrolyte solution [2]. One of the most promising electrode geometries is interdigitated arrays of microelectrodes (IDEs), mainly in terms of detection sensitivity and the possibility for signal amplification [4]. The two fingers of the IDEs can be biased at the same or at different potentials depending on the application [5], rendering the platform very versatile.

Regarding the choice of material, a great number of applications utilize gold WEs as they are easy to functionalise, e.g., by thiols, and relatively inert [6–8]. Typical REs are composed of Ag/AgCl with an internal electrolyte (KCl) and therefore their integration in microsensor devices is cumbersome and requires additional microfluidic channels and complex microfabrication steps. Due to the complexity of these designs there is usually only one measuring site per chip, which restricts the possible applications of these devices for lab-on-a-chip and especially point-of-care systems, where multiple measurements on a single sample are often needed. Furthermore, using an Ag/AgCl electrode might compromise the biocompatibility of the chip, if used for electrochemical detection from cell cultures or *in vivo*. Over long-term exposure to culturing media or physiological conditions, a possible release of Ag^+^ ions and degradation of the electrode surface materials can occur [9]. For these reasons, Au is a preferred REs material when it comes to microsensors for biological applications. Moreover, gold electrodes have been extensively characterized and their electrochemical behavior is well known, not only for WEs, but also for the cases of REs and CEs.

Although gold films are easily integrated into microfabrication processes, e.g., lift-off techniques and other micro-lithography based processes, there are still major issues related to gold electrode fabrication that need to be addressed.

Metal lift-off, based on the removal of the excess gold by dissolving the resist on which the gold is deposited, is one of the most popular methods for creating gold electrodes due to its simplicity. A resist undercut is required in order not to create a continuous gold film so that lift-off is easier and negative tone resists or a chemical treatment of a positive tone resist can be used for the purpose [10]. However, a common problem with this technique is the appearance of ear-like structures at the edges of the gold (or other metal) patterns (known as “rabbit ears” or “lift-off ears”). The presence of lift-off ears renders additional microfabrication steps cumbersome and often impossible; this is particularly the case when a thin film layer has to be deposited on top of these structures, as is the case in this work. Moreover, the sharp edges mean that a powerful electric field can be generated there, which can disrupt measurements involving electric fields in liquids. Therefore a reliable method for avoiding their formation is imperative.

In order to monitor electrochemical reactions only at the electrodes and not also on the connecting wires, most of the chip's surface needs to be insulated, leaving openings only at the active electrode surfaces (and the contact pads). Materials used for the purpose can be dielectrics, e.g., silicon oxide and silicon nitride, or polymers, e.g., SU8 or polyimide. Silicon oxide and silicon nitride layers are preferred because of the ability to create thin layers and the ease in integration with other microfabrication process steps. The deposition of these materials is also well known and characterized in cleanroom processes. Good adhesion between the passivation material and the surface of the metal is needed in order to avoid creeping of liquid under the passivation layer. This can be an issue in itself for both silicon oxide [11] and silicon nitride but more so in relation to the existence of lift-off ears, which inevitably will have an effect on adhesion.

In this paper we present a versatile and reproducible fabrication process for a microchip design having 12 identical sets of microelectrodes, each including an interdigitated WE, one RE and one CE, for use in various bioanalytical applications relying on electrochemical detection. We show how the effect of lift-off ears can be avoided by using an additional etching step before the metal deposition and how the use of a 500 nm thick layer of silicon nitride improves the stability and functionality of the passivation layer. The influence of the optimized fabrication process and electrode cleaning on high reproducibility of electrochemical processes is demonstrated using cyclic voltammetry (CV) and electrochemical impedance spectroscopy (EIS). Finally, examples of the functionality of the microchips in DNA hybridization and protein nanofibril-based glucose biosensing applications are shown.

## 2. Experimental Section

### 2.1. Chip Design and Fabrication Process

The chip dimension was determined so that the number of chips patterned on a 4-inch wafer could be maximized to keep the fabrication costs sufficiently low. Due to this, the counter electrode to working electrode area ratio was decreased to 6.4 (for each side of the interdigitated electrodes) although a ratio of at least 10 would be more optimal. We also introduced a simple pseudo reference electrode on chip, fabricated simultaneously with the working and counter electrodes and show that this does not affect the electrochemical performance of the chip, even in applications where the reference electrode is modified during electrode preparation or measurements. The distances between the WE and CE and RE and WE were chosen in order to maximize the number of individual measuring sites and not after consideration of the IR drop; however, due to the small distances and the size of the WE the IR drop is generally small [12].

The microchip design was based on specific requirements regarding the number of desired individual measuring sites, the size of the electrodes in each measuring site, the space occupied by contact pads, on-chip wiring to the electrodes and space for integrating an individually addressable microfluidic electrochemical cell to each measuring site. Moreover, the on-chip wiring to the electrodes was designed to be symmetric in order to simplify the construction of a miniaturized potentiostat to be integrated on a microfluidic platform holding the microchip for electrochemical measurements [5]. An additional design constrain was that cleanroom fabrication rules limited the use of metal to a maximum of 8%–9% of the surface of a wafer. The design also allows for the fabrication of 12 microfluidic chambers connected by a smaller number of microfluidic channels to inlets and outlets on the chip. The microfluidic system will not be addressed in this paper.

**Figure 1.**
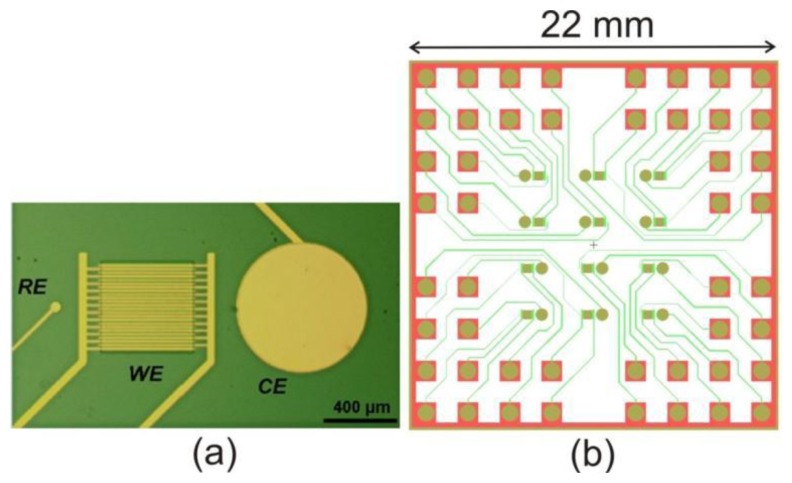
(**a**) Optical microscopy image of a single measuring site with RE, CE and two working electrodes comprising an IDE, taken after the end of the fabrication process; (**b**) Layout of the microchip containing 12 measuring sites.

The width of and gap between the interdigitated electrodes (IDEs) was set to be 10 µm, although the fabrication process was also tested on structures of 2, 3 and 5 µm (width was always equal to the gap size). Each microchip contains 12 identical electrochemical measuring sites, each comprising an IDE, a reference electrode with a diameter of 50 µm and a counter electrode with a diameter of 700 µm. The sizes of the RE and CE were chosen in order to satisfy space requirements in each measuring site and the need of having a CE with significantly larger surface area than the IDE [13]. An individual set of IDE, RE and CE can be seen in Figure 1a. To accommodate all the electrodes and the on-chip wiring, each microchip has the dimensions of 22 × 22 mm^2^, facilitating fabrication of nine chips on a 4-inch wafer. The layout of a microchip is shown in Figure 1b.

**Figure 2.**
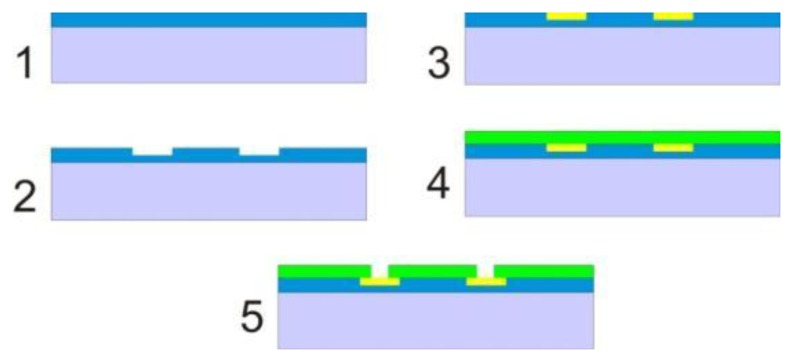
Summary of the optimised fabrication process. (**1**) 500 nm silicon dioxide are deposited on a silicon wafer; (**2**) The electrodes are patterned by photolithography and a HF dip creates undercuts at the locations where the metals are to be deposited; (**3**) 10 nm Ti and 150 nm Au are deposited by a thermal evaporation process and the electrodes are formed by lift-off; (**4**) 500 nm of silicon nitride are deposited by PECVD; (**5**) The nitride layer is patterned by photolithography and etched with RIE to open the electrode areas and the contact pads.

The optimised fabrication process is schematically summarized in Figure 2. First, 500 nm of silicon dioxide were thermally grown on a standard single-polished silicon wafer in a drive-in furnace at 1050 °C. All the metal structures, *i.e.*, electrodes, wiring and contact pads, were defined by photolithography using a positive photoresist (AZ^®^ 5214E from MicroChemicals GmbH, Ulm, Germany) with an image reversal process. After development the wafer was dipped in HF for 100 s in order to form a 150 nm deep isotropic undercut into which the metals (10 nm Ti and 150 nm Au) were deposited by electron beam evaporation. The HF dip was necessary in order to avoid the formation of lift-off ears at the edges of the metallic structures, as is shown in the results and discussion section. The structures were subsequently formed after lift-off in acetone. All non-active gold areas (the wiring from the electrodes to the contact pads) were passivated by 500 nm silicon nitride deposited by plasma-enhanced chemical vapour deposition (PECVD). Etching of the nitride from the active gold areas was done after another photolithographic step, again using an image reversal process, coupled with a reactive ion etch (RIE). The photoresist used as the etching mask was removed by ultrasonication in acetone followed by intermediate rinsing with ethanol and final rinsing with deionised water.

During the optimization of the fabrication process we also tested the use of a 1 µm thick PECVD deposited silicon dioxide layer as the passivation layer. A comparison of the two materials as passivation layers is presented in the Results and Discussion section.

### 2.2. Atomic Force Microscopy Measurements

A Nanoman Atomic Force Microscope (AFM) from Bruker Corporation (Billerica, MA, USA) was used in order to characterize the height of the lift-off ears at the edges of the metallic structures (mostly the IDEs). Images were taken at several positions on the wafer in order to determine whether the chip location on the wafer had an effect on the measured height.

### 2.3. Electrochemical Characterization

Prior to the electrochemical characterization the electrode chips were cleaned by treatment with a solution composed of 50 mM KOH and 25% H_2_O_2_ for 10 min, followed by a potential sweep of the IDEs from −0.2 to −1.2 V in 50 mM KOH at the potential scan rate of 50 mV/s to remove the gold oxides formed during the chemical cleaning [14].

To characterize the effects of the cleaning procedure on the electrode chips both electrochemical impedance spectra (EIS) and cyclic voltammograms (CVs) were acquired using an Autolab potentiostat (PGSTAT302N, Utrecht, The Netherlands) before and after every cleaning step in a solution containing 10 mM of ferri/ferrocyanide ([Fe(CN)_6_]^3^**^−^**^/4^**^−^**) in phosphate buffer saline (PBS). For EIS recordings a sinusoidal perturbation potential (10 mV RMS *vs.* the open circuit potential) was applied between the on-chip CEs and IDEs of each measuring site in the frequency range between 200 mHz and 1 MHz. The CVs were recorded at the scan rate of 100 mV/s in the potential window from −0.5 to 0.5 V.

To demonstrate that all of the 12 electrode sets on a chip are identical, CVs were acquired on each of the IDEs (the same electrolyte solution as above) using a CH1000A multichannel potentiostat (CH Instruments Inc., Austin, TX, USA). A potential scan rate of 100 mV/s was used in the potential window from −0.35 to 0.35 V. The on-chip RE and CE of each measuring site were used for the CV recordings. For EIS spectra showing the similarity between the 12 electrode sets the same parameters as for the characterization were used.

Additionally, the DNA hybridization on the IDEs of the electrode sets was monitored with EIS using an Autolab PGSTAT302N potentiostat. Prior to DNA sensing the electrodes were cleaned as described above; however, the potential cycling was performed from −0.2 to −1.4 V. The chips were modified by overnight incubation in a humidity chamber at room temperature (RT) with a 1 µM thiol modified DNA probe. After washing in PBS the chips were incubated with 1 µM complementary DNA solution in PBS for 1 h at RT. Before the measurements the chips were washed in PBS. All DNA sequences used in the project are listed in Table 1.

The EIS recordings were done using the same parameters and electrolyte solution as described above. The impedance spectra were acquired after each step of the DNA sensing procedure: (1) on bare cleaned gold electrodes; (2) after overnight immobilization of SH-DNA probe; and (3) after hybridization with a complementary DNA (cDNA) target. As all of the electrodes were modified with the DNA probe (*i.e.*, also the reference electrode), impedance spectra acquired using the on-chip RE of each measuring site were compared with spectra acquired using an external custom made pseudoreference electrode (a silver wire covered with AgCl) placed in the measurement chamber. Moreover, a control experiment with non-complementary DNA was carried out to evaluate the source of the signal change.

Protein nanofibrils (PNFs) from whey protein isolate were fabricated and functionalized with glucose oxidase and thiol moieties using a previously reported multifunctionalization approach [15]. Deposition of the functionalized PNFs onto electrodes was done by pipetting 200 µL of a PNF solution (2 mg/mL) onto the microchip and allowing the PNFs to sediment onto the electrodes for 30 min. After washing of the microchip with milliQ water, the cyclic voltammetry measurements were carried out using a mediator solution of potassium ferricyanide ([Fe(CN)_6_]^3−^, 10 mM) with and without the inclusion of d-glucose (100 mM). The mediator concentration was chosen after an optimization process to clearly visualize the detection peak. A control experiment using fibrils that had not been functionalized with glucose oxidase was also conducted.

## 3. Results and Discussion

### 3.1. Fabrication

The fabrication process used for the electrodes had three requirements: (1) The gold electrodes fabricated should be relatively equal in terms of width and height across the wafer; (2) The width of and the gap between the digits of the IDEs should be equal; (3) The passivation layer should adhere well on the substrate (both the oxide and the gold electrodes) so that creeping of electrolyte under the layer and onto the metal leads could be eliminated. In an image reversal process an undercut profile is expected to be formed in the developed photoresist which prohibits the deposition of a continuous metal film. Apart from being difficult to lift-off, such a continuous film would also create rough edges (lift-off ears) on the metallic structures, which are unwanted in a process aiming at the fabrication of robust well-defined electrode areas.

#### 3.1.1. Elimination of Lift-Off Ears

**Figure 3.**
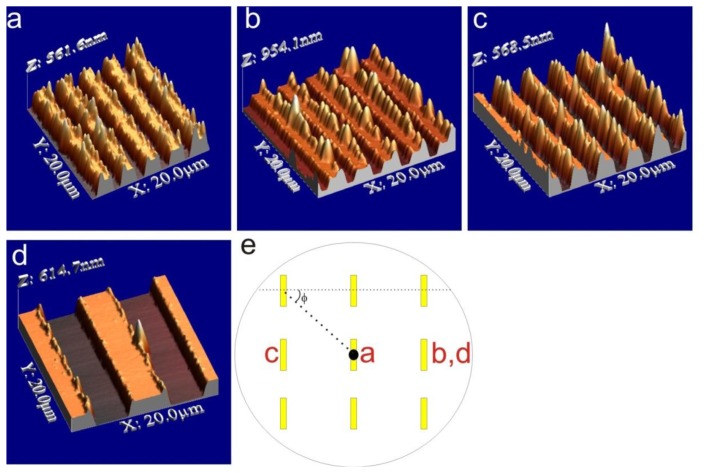
3D reconstruction of AFM images showing the formed lift-off ears. (**a**) 2 µm width structures at the middle of the wafer; (**b**) 2 µm width structures at one edge of the wafer; (**c**) 2 µm width structures at the opposite edge of the wafer relative to (b); and (**d**) 5 µm width structures at the right side of the wafer; (**e**) Schematic of the metal evaporation process that is thought to cause the uneven distribution of the height of the lift-off ears. The letters mark the location at which the images (a)–(d) were taken.

To confirm that no edges were present AFM characterization of the IDEs was performed after the lift-off process. The obtained results are shown in Figure 3 and were contrary to expectations. Despite the applied image reversal process lift-off ears were formed on the electrode structures, with heights ranging from 10 nm all the way to 900 nm above the electrode surface. The height of the lift-off ears was depending on the electrode width and the location of the electrode chip on the wafer. Figure 3a–c show 2 µm wide structures from different parts of the wafer, while Figure 3d shows 5 µm wide structures. Although lift-off ears were also present on the 5 µm wide structures, they were considerably lower, with height distribution up to 200 nm, which still is, however, of the same order of magnitude as the height of the electrodes.

It is clear from Figure 3 that the location of the electrode chips with respect to the centre of the wafer has an effect on the height of the ears. Since thermal evaporation was used, the metal is deposited on the wafer from a point source (where the electron beam hits the metal target in the cubicle) following line of flight, thus hitting a particular point on the wafer at an angle dependent on the distance of the point from the centre of the wafer (see illustration in Figure 3e). This means that for electrodes located at the edges of the wafer, one side of the photoresist is covered with more metal than the other side, which results in the formation of higher lift-off ears. Furthermore, the angle *φ* of the electrodes relative to the incoming trajectory of the metal also influences the height distribution of the formed lift-off ears: An angle of 0° results in the most one-sided deposition whereas at an angle of 90° the ear formation is more even. The deposition angle cannot be fully controlled during metal evaporation, since placement of the wafer on the holder is done manually. Due to this, the effect of the deposition angle was only noted after most of the wafer batches had been processed. However, it was clear that the electrodes located closest to the edges of the wafer had the most uneven height distribution of lift-off ears. It should be noted that the inhomogeneity of the ear height could to some extent be compensated by rotating the holder during deposition. In that case the deposition would have been more homogeneous across the wafer, but the lift-off ears would not be eliminated.

**Figure 4.**
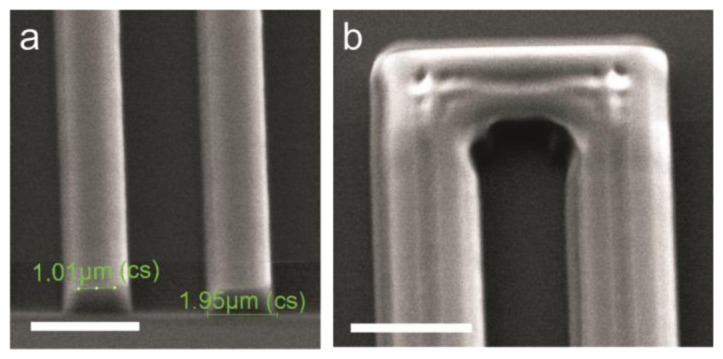
SEM images (at 30° tilt) of the resist structure. (**a**) The 2 µm structures (resist lines represent the area between the electrodes), showing an almost 2 fold difference in the width of the top and bottom layer of the resist, with a narrower top part. The scalebar is 3 µm; (**b**) The resist structure at the end of an electrode, showing an inhomogeneous surface topography with a central groove. The electrode (dark region in between the light regions) is also ill-defined at the corner. The scale bar is 5 µm.

Despite considerable lithography optimization in terms of exposure time and exposure mode (constant intensity or constant power) the ears were present every time a new wafer batch was started. Moreover, the width of and gap between the IDEs was not the same for the smaller dimensions, having variation by as much as 25%. Although a small variation is to be expected, given the nature of photolithography, this large deviation cannot only be explained by this. After having first examined the used lithographic mask, the developed photoresist was imaged by Scanning Electron Microscopy (SEM). The results, shown in Figure 4, confirm that the photolithography process is the probable cause for the aberrations in electrode width and gap: (a) the resist profile does not have an undercut, but the resist is broader at the bottom of the layer and narrower at the top; and (b) the top surface of the resist is not homogeneous in height but has grooves that seem to follow the structures on the mask. The results presented in Figures 3 and 4 appeared continuously over a period of 1 year for eight batches of wafers fabricated by two cleanroom trained researchers, an experienced cleanroom technician and a lithography process specialist. During the same period no other lithography problems were reported by cleanroom users. Therefore, we concluded that the problem may have been caused by lightwave interference due to the geometry of the fabricated structures.

The formation of the ears, a common challenge in lift-off processes, poses a large problem for the further passivation of the electrodes by PECVD oxide or nitride, and therefore its removal or avoidance is imperative. We investigated the use of iodine etch (100:25:500 KI:I_2_:H_2_O (w/w/v)) to remove the sharp ears, which are expected to be etched much faster than the bulk of the gold electrodes. However, the etch rate of gold was not controllable using this approach; even a few seconds of exposure to the etch solution removed entirely the gold structures from the wafer. For the same reason it was not possible to fabricate the electrodes by first depositing the metal followed by patterning the electrodes through gold etching.

**Figure 5.**
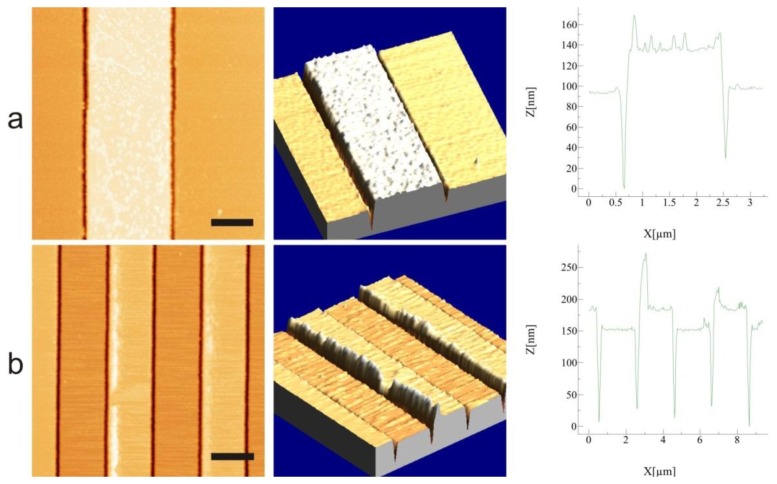
AFM images of 2 µm wide electrodes using the HF etching technique prior to metallization. (**a**) An electrode where no ears are present; and (**b**) an electrode with existing ears with a height that is considerably lower than before and with only partial coverage of the electrode surface. The left panels show the AFM image, the middle panels show a 3D reconstruction of the AFM image and the right panels show a line profile across the electrode width. Scale bar is (a) 1 µm and (b) 2 µm.

A solution to the problem was found by introducing an HF etching step after the photolithography of the electrodes prior to depositing the metals [16,17]. Due to the isotropic nature of the HF etch, a groove on the silicon dioxide surface is created, which is slightly wider than the area where the resist is removed. By timing the HF etch so that about 150 nm of silicon dioxide are removed, only a few nanometers of metal remain over the silicon dioxide surface. AFM images of the resulting structures after metal deposition and lift-off are shown in Figure 5. By utilizing this HF etching step a significant decrease in the formation of ears is achieved both in terms of electrode area coverage as well as ear height. As shown in Figure 5b, the maximum ear height is slightly over 50 nm compared to the previously observed heights of over 900 nm. Moreover, based on optical microscopic examination of the structures, we estimate that when using the HF technique maximally 10% of the electrode area has lift-off ears, compared to a nearly total coverage when no etching is used.

#### 3.1.2. Optimization of Passivation Layer

**Figure 6.**
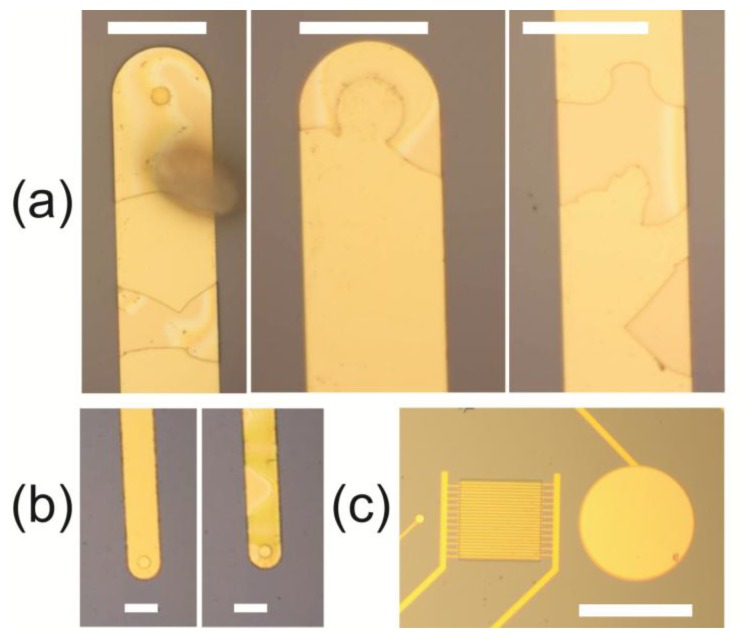
(**a**) Optical microscope images of electrodes and leads with visible cracks in the silicon oxide passivation layer; (**b**) The reference electrode before (left) and after (right) a CV was acquired. New cracks are visible after acquisition of the CV; (**c**) The three electrodes after fabrication, wafer dicing, cleaning and measurement. No cracks can be seen in the utilized silicon nitride passivation layer. Scale bars in (a) and (b) are 50 µm, and in (c) 700 µm.

Passivation of the electrodes was originally attempted by depositing 1000 nm of PECVD silicon dioxide. The effect of the lift-off ears on the passivation layer can be seen both immediately by optical microscopy after finishing the fabrication process and electrochemically after acquiring a CV on the fabricated electrode chips. Cracks are present on the silicon dioxide layer and there are entire regions with no silicon dioxide at all (Figure 6a). These missing pieces are found deposited on other parts of the wafer. Moreover, the oxide layer that does adhere on the metallic leads after the finished fabrication process cracks during acquisition of a CV due to the large electric field generated at the very sharp edges of the ears (Figure 6b).

On the chips with no (or with limited presence of) lift-off ears, *i.e.*, those fabricated with the HF etching step, there are no cracks present after the last fabrication step. These are however introduced on the larger structures during dicing of the wafers with a diamond saw in order to acquire the individual electrode chips. This can be avoided to a certain extent by dicing the wafers before removing the photoresist layer used in the last lithography step for patterning the silicon oxide passivation layer. In this case, the resist is removed outside the cleanroom on a chip-to-chip basis by soaking each chip in boiling acetone. Acquisition of CVs does not introduce cracks in this case; however it was noticed that the cleaning procedure before electrochemical characterization also can introduce cracks in the passivating oxide layer.

For these reasons the passivation layer was changed to PECVD silicon nitride, which adheres better on the gold electrodes and is commonly used as a passivation layer [18]. Although nitride takes longer time to deposit on the wafer and also increases the parasitic capacitance of the chip, the etching time in RIE for the 500 nm layer is only 7 min, compared to about 1 h for the PECVD oxide, and there are no visible cracks after the fabrication, dicing of the wafers, removal of the resist or after measurements (Figure 6c). The adhesion of the nitride to the gold electrodes can further be quantified by looking at the EIS spectra acquired during the characterization, as will be presented in the next section. PECVD silicon nitride is generally free of pinholes (less than 1 pinhole per cm^2^ [19]) and according to the cleanroom quality control data the number of particles on the nitride, the refractive index and the deposition rate were all within the specifications at the time of the fabrication. No further measurements of the pinhole density were therefore carried out. Finally, electrical breakdown measurements for the deposited nitride were carried out at the non-etched regions by sweeping the voltage across the nitride layer from 0 to 200 V (limit of the device) without any measurable breakdown of the nitride. The current through the nitride at 200 V was about 180 nA.

### 3.2. Electrochemical Characterization

Considering that the best results in terms of electrode dimension reproducibility, ear removal and passivation were achieved with larger size structures the chip design was modified by changing the electrode width to 10 µm. All characterization results presented in this section were obtained on the IDEs having 10 µm width and gap size.

**Figure 7.**
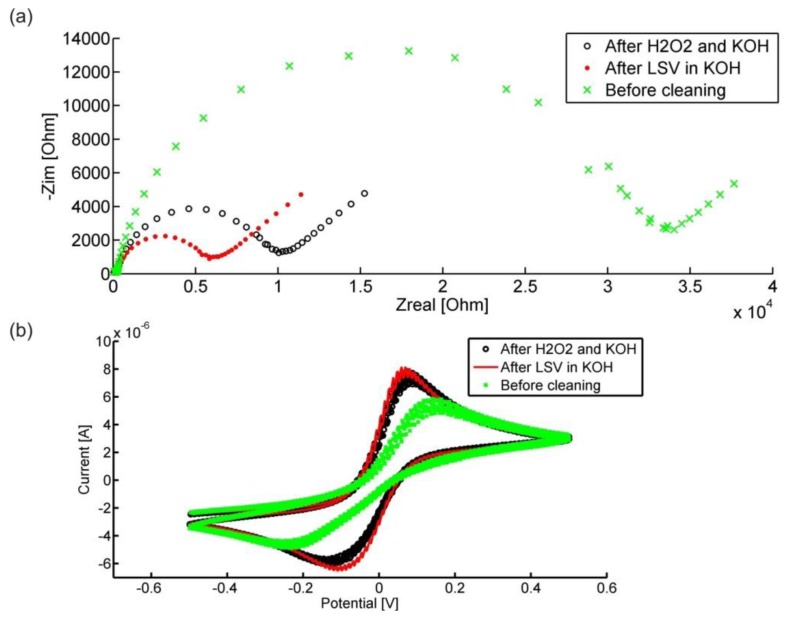
(**a**) Impedance spectra and (**b**) CVs recorded on a WE before and after each cleaning step. Each step reduces the charge transfer resistance and improves the cathodic and anodic peak of the CVs.

The effect of the cleaning process can be seen in Figure 7. Impedance spectra (Figure 7a) and CVs (Figure 7b) acquired on the IDEs are shown both before cleaning and after the two cleaning steps. The impedance spectra show how the charge transfer resistance is reduced after each cleaning step and the CVs show how the cathodic and anodic peaks become more pronounced. Usually there is only a small difference between the CVs after the 1st and 2nd cleaning steps, which is in agreement with the results described in [14].

The behavior shown in Figure 7 is reproducible on all 12 IDEs of a chip, although the quality of the electrodes before the cleaning steps can sometimes vary significantly. The reason for this is most likely the dicing of the wafers to obtain the individual chips and the subsequent removal of the resist after the dicing. Dicing introduces a significant amount of silicon particles on the wafer surface (and thus on the electrodes) and the resist cannot be removed effectively by only soaking in boiling acetone. Therefore it is plausible that directly after fabrication some of the electrodes are cleaner than others.

**Figure 8.**
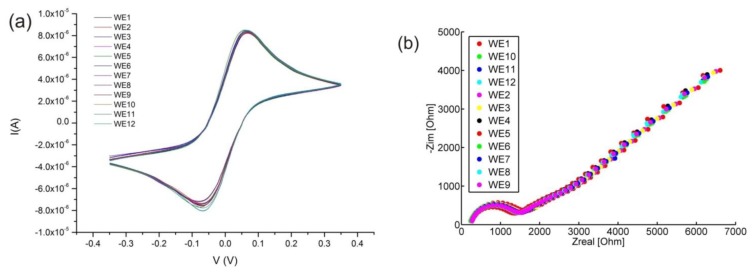
CVs (**a**) and EIS spectra (**b**) recorded on all of the 12 WEs using the on chip reference and counter electrodes for each measuring site. The CVs and EIS spectra are well superimposed on each other, presenting practically identical peak potentials and currents as well as impedance on each of the 12 electrodes.

After the cleaning described in the materials and methods section all of the 12 sets of electrodes are practically identical in their electrochemical behaviour. This can be seen in Figure 8a, showing CVs acquired on all of the 12 electrodes. The 12 CVs are significantly superimposed on each other, confirming the good reproducibility of both the fabrication process and the cleaning procedure. EIS spectra acquired on all of the 12 electrodes after cleaning are also identical (Figure 8b).

Figure 8b also provides evidence of the good adhesion between the nitride passivation layer to the gold electrodes. In the case of bad adhesion, *i.e.*, a cavity forming between the metal and the passivation layer, there would be a low frequency distortion of the spectra. This means that the straight line of Figure 8b at roughly an angle of 45° would no longer be a line but a curve. This is indeed the case when EIS spectra is taken for oxide as a passivation layer (data not shown).

### 3.3. Applications of the Microelectrode Array Chip

**Figure 9.**
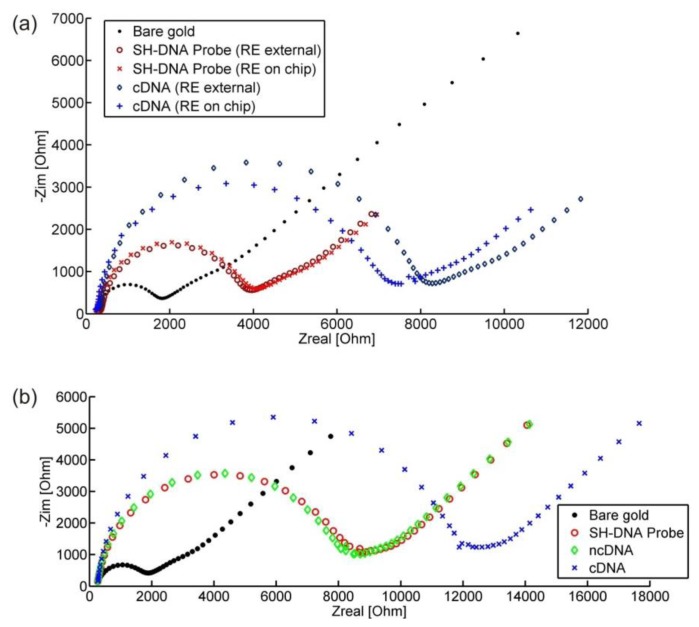
(**a**) Impedance spectra acquired on a bare WE, the same WE after modification of the electrode surface with a DNA probe and after hybridization with a complementary DNA (cDNA) using the on chip and an external reference electrode. The difference between spectra recorded with the two different REs is minimal after the first modification but is increased when the surface of the on-chip reference electrode is further modified by the cDNA. However, detection of DNA hybridization is clearly possible using the on-chip RE; (**b**) Control experiment (using RE on chip) where a non-complementary DNA strand is first hybridized on chip. The EIS spectra remain virtually unchanged as no binding occurs between the DNA probe and the non-complementary strand.

Many applications of the electrodes involve modification of their surface using for instance deposition of self-assembled monolayers (SAMs) of thiols [20]. In such processes all the gold electrodes, including REs and CEs are modified simultaneously although only the IDEs should ideally be modified. This can inevitably influence the recorded electrochemical signal. We have therefore compared the sensing abilities of the electrodes in terms of monitoring DNA hybridization when using the on-chip RE and an external pseudo RE. After incubation with a DNA probe the charge transfer resistance (the semicircle of Figure 9a) increases, as the DNA probe is negatively charged and therefore inhibits the electrons from the mediator reaching the electrode. When complementary DNA binds to the DNA probe the negative charge on the electrode increases further, as does the recorded charge transfer resistance. No such change is observed when the DNA is a non-complementary strand, as can be seen in Figure 9b, as there is no binding with the immobilized DNA probe occurring. We note that the data presented in Figure 9a,b are not from the same experiment; the change in charge transfer resistance depends on the amount of the probe/complementary DNA binding on the chips, which can differ from experiment to experiment.

As can be seen in Figure 9a, there is a difference in the recorded impedance spectra after DNA hybridization when using either the on-chip RE, which is modified together with the IDEs, or an external RE. Although there is a difference between the spectra recorded with the two different REs, this is quite small and the detection of the hybridization is still clearly possible using the on-chip REs. Our results are comparable with those obtained using an external reference electrode [21] or an on-chip reference electrode made of platinum [22].

**Figure 10.**
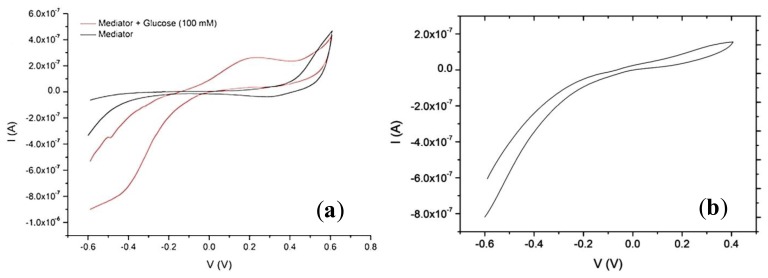
(**a**) Typical cyclic voltammogram of the biosensor platform's response to glucose (100 mM) in a Ferricyanide mediator (10 mM) (red) and to the mediator alone (black), using individual WE/CE/RE microelectrode sets and a potential sweep rate of 100 mV/s; (**b**) A CV obtained from electrodes modified with fibrils without the immobilized glucose oxidase enzyme.

An additional application for the microelectrodes created in this work is the creation of a glucose-biosensing platform. PNFs formed from whey protein isolate were utilized as a nanoscaffold for glucose oxidase (GOx) immobilization on the interdigitated WEs. The PNFs were functionalized with GOx for biosensing and with thiol moieties for gold surface attachment using a multifunctionalization approach [15]. After PNF-GOx immobilization onto the electrodes, cyclic voltammetry was used as detection method. The mediated glucose response is seen by an increase in anodic peak current (Figure 10a). The glucose detection peak is reflecting the mediator oxidation upon the redox reactions occurring between the glucose and the glucose oxidase immobilized on the electrodes via the protein nanofibrils. There is a small reduction peak (both in the presence and not of glucose), but it is hidden by a large cathodic ohmic behavior which arises due to the nature of the electrode's reversibility characteristics in this particular batch and due to the high double-layer capacitance from the presence of the protein nanofibrils (and other protein material) sitting on the surface of the electrode. As a control, the response of electrodes covered with non-modified fibrils (*i.e.*, fibrils without the glucose oxidase enzyme) and exposed to the same detection solution (glucose + mediator) was recorded and shown in Figure 10b. There is again a large cathodic current as before but no oxidation peak can be seen. Although there are some issues with the electrode surface properties in this particular experiment, there is a clear difference between the control and the glucose oxidase modified nanofibrils. These results are comparable to similar PNF-based glucose biosensor systems that utilize commercially available electrodes [15].

## 4. Conclusions/Outlook

In conclusion, we have presented a simple and robust process for the fabrication of microelectrode chips having 12 identical individually addressable electrochemical measuring sites. The common microfabrication issue of the lift-off ears has been solved by an HF dip before metallization and the electrodes have been effectively passivated by a 500 nm silicon nitride layer exposing only the small electrode areas to the analytes and thus reducing measuring noise. The optimized protocol has been used in processing numerous batches of wafers during the past 3 years with consistent and reproducible results, yielding WEs having the same impedance levels after the optimized cleaning procedure.

The chips have been characterized by cyclic voltammetry and electrochemical impedance spectroscopy and the results show that all 12 electrodes are identical in their electrochemical behaviour. Moreover, we have shown that the on-chip reference electrode can be used even when the application calls for a modification of the surface of the electrodes, which was successfully demonstrated by the detection of DNA hybridization by impedance spectroscopy and by a protein nanofibril-based glucose biosensing via cyclic voltammetry.

The fabricated chips have been used in various applications, such as monitoring of dopamine exocytosis from PC12 cells, where the electrodes were modified by an overoxidised polypyrrole layer [23] and mercaptopropionic acid SAM [24].

Finally, we note that the chip design is easily modified in order to accommodate other electrode geometries, like for example a 7 × 7 array of circular flat gold or 3D electrodes in polymer [25] centered on the chip. Furthermore, by adding a few extra steps in the fabrication sequence to accommodate for patterning and etching structures in silicon by KOH, arrays of 3D electrodes [26,27] can be made on the same platform, thus improving the electrode impedance due to the increased surface area [28].

## Figures and Tables

**Table 1.** Details of the DNA sequences used in the DNA experiments.

**Name**	**Sequence**
DNA probe (SH-DNA)	5′-SH-C6-TGTACATTTTCTTATATTCT-3′
Complementary DNA (cDNA)	5′-AGAATATAAGAAAATGTACATGTCCTGTTTTCTAAATTGT-3′
Non-complementary DNA (ncDNA)	5′-CGGGATACAATTGGTGTTGGACTTTTAAAAATAGCTTTTA-3′

## References

[1] Nyholm L. (2005). Electrochemical techniques for lab-on-a-chip applications. Analyst..

[2] Grieshaber D., MacKenzie R., Voros J., Reimhult E. (2008). Electrochemical biosensors—sensor principles and architectures. Sensors.

[3] Li H.Q., Luo X.B., Liu C.X., Jiang L.Y., Cui D.F., Cai X.X., Yang Q.D. Multi-Channel Electrochemical Detection System Based on Labview.

[4] Cohen A.E., Kunz R.R. (2000). Large-area interdigitated array microelectrodes for electrochemical sensing. Sens. Actuators B Chem..

[5] Vergani M., Carminati M., Ferrari G., Landini E., Caviglia C., Heiskanen A., Comminges C., Zor K., Sabourin D., Dufva M. (2012). Multichannel bipotentiostat integrated with a microfluidic platform for electrochemical real-time monitoring of cell cultures. IEEE Trans. Biomed. Circuits Syst..

[6] Lucarelli F., Marrazza G., Turner A.P.F., Mascini M. (2004). Carbon and gold electrodes as electrochemical transducers for DNA hybridisation sensors. Biosens. Bioelectron.

[7] Zhang S., Wright G., Yang Y. (2000). Materials and techniques for electrochemical biosensor design and construction. Biosens. Bioelectron.

[8] Li G., Miao P. (2012). Electrochemical Analysis of Proteins and Cells.

[9] Moussy F., Harrison D.J. (1994). Prevention of the rapid degradation of subcutaneously implanted ag/agcl reference electrodes using polymer coatings. Anal. Chem..

[10] Lee H.S., Yoon J.-B. (2005). A simple and effective lift-off with positive photoresist. J. Micromech. Microeng..

[11] George M.A., Bao Q.C., Sorensen I.W., Glaunsinger W.S., Thundat T. (1990). Thermally induced changes in the resistance, microstructure, and adhesion of thin gold films on si/sio [sub 2] substrates. J. Vac. Sci. Technol. A Vac. Surf. Films.

[12] Huang X.-J., O'Mahony A.M., Compton R.G. (2009). Microelectrode arrays for electrochemistry: Approaches to fabrication. Small.

[13] Brett C.M.A., Brett A.M.O. (1993). Electrochemistry: Principles, Methods, and Applications.

[14] Fischer L.M., Tenje M., Heiskanen A.R., Masuda N., Castillo J., Bentien A., Emneus J., Jakobsen M.H., Boisen A. (2009). Gold cleaning methods for electrochemical detection applications. Microelectron. Eng..

[15] Sasso L., Suei S., Domigan L., Healy J., Nock V., Williams M.A.K., Gerrard J.A. (2014). Versatile multi-functionalization of protein nanofibrils for biosensor applications. Nanoscale.

[16] Reimer K., Kohler C., Lisec T., Schnakenberg U., Fuhr G., Hintsche R., Wagner B. (1995). Fabrication of electrode arrays in the quarter micron regime for biotechnological applications. Sens. Actuators A Phys..

[17] Tong H.D., Zwijze R.A.F., Berenschot J.W., Wiegerink R.J., Krijnen G.J.M., Elwenspoek M.C. Characterization of Platinum Lift-Off Technique.

[18] Xu Q., Ra Y., Bachman M., Li G.P. (2009). Characterization of low-temperature silicon nitride films produced by inductively coupled plasma chemical vapor deposition. J. Vac. Sci. Technol. A..

[19] Stoffel A., Kovács A., Kronast W., Müller B. (1996). Lpcvd against pecvd for micromechanical applications. J. Micromech. Microeng..

[20] Silva M.S., Cavalcanti I., Barroso M.F., Sales M.G., Dutra R. (2010). Gold electrode modified by self-assembled monolayers of thiols to determine DNA sequences hybridization. J. Chem. Sci..

[21] Keighley S.D., Li P., Estrela P., Migliorato P. (2008). Optimization of DNA immobilization on gold electrodes for label-free detection by electrochemical impedance spectroscopy. Biosens. Bioelectron.

[22] Ben-Yoav H., Dykstra P.H., Bentley W.E., Ghodssi R. (2012). A microfluidic-based electrochemical biochip for label-free diffusion-restricted DNA hybridization analysis. Biosens. Bioelectron.

[23] Sasso L., Heiskanen A., Diazzi F., Dimaki M., Castillo-Leon J., Vergani M., Landini E., Raiteri R., Ferrari G., Carminati M. (2013). Doped overoxidized polypyrrole microelectrodes as sensors for the detection of dopamine released from cell populations. Analyst.

[24] Zor K., Vergani M., Heiskanen A., Landini E., Carminati M., Coman V., Vedarethinam I., Skafte-Pedersen P., Skolimowski M., Martinez Serrano A. Real-time monitoring of cellular dynamics using a microfluidic cell culture system with integrated electrode array and potentiostat.

[25] Abaddi M.A., Sasso L., Dimaki M., Svendsen W.E. (2012). Fabrication of 3d nano/microelectrodes via two-photon-polymerization. Microelectron. Eng..

[26] Dimaki M., Vazquez P., Aimone A., Olsen M.H., Sasso L., Rodriguez-Trujillo R., Svendsen W.E. (2012). Novel 3d microelectrodes and pipettes by wet and dry etching. Microelectron. Eng..

[27] Dimaki M., Vazquez P., Olsen M.H., Sasso L., Rodriguez-Trujillo R., Vedarethinam I., Svendsen W.E. (2010). Fabrication and characterization of 3d micro- and nanoelectrodes for neuron recordings. Sensors.

[28] Vazquez P., Dimaki M., Svendsen W.E. Scalloped electrodes for highly sensitive electrical measurements.

